# Novel Chimeric Multiepitope Vaccine for Streptococcosis Disease in Nile Tilapia (*Oreochromis niloticus* Linn.)

**DOI:** 10.1038/s41598-019-57283-0

**Published:** 2020-01-17

**Authors:** Ansaya Pumchan, Sucheewin Krobthong, Sittiruk Roytrakul, Orathai Sawatdichaikul, Hidehiro Kondo, Ikuo Hirono, Nontawith Areechon, Sasimanas Unajak

**Affiliations:** 1 Department of Biochemistry, Faculty of Science, Kasetsart University, 50 Ngam Wong Wan, Chatuchak, Bangkok 10900 Thailand; 2 Proteomics Laboratory, Genome Institutes, National Center for Genetic Engineering and Biotechnology, Pathumthani, 12120 Thailand; 3 Department of Nutrition and Health, Institute of Food Research and Product Development, Kasetsart University, 50 Ngam Wong Wan, Chatuchak, Bangkok 10900 Thailand; 4 Graduate School of Marine Science and Technology, Tokyo University of Marine Science and Technology, Konan 4-5-7, Minato-KU, Tokyo, 108-8477 Japan; 5 Department of Aquaculture, Faculty of Fisheries, Kasetsart University, 50 Ngam Wong Wan Road, Chatuchak, Bangkok 10900 Thailand; 6 Omics Center for Agriculture, Bioresources, Food and Health, Kasetsart University (OmiKU), Kasetsart University, 50 Ngam Wong Wan Road, Chatuchak, Bangkok 10900 Thailand; 7 Center for Advanced Studies for Agriculture and Food, KU Institute for Advanced Studies, Kasetsart University, (CASAF, NRU-KU, Thailand), Bangkok, 10900 Thailand

**Keywords:** DNA vaccines, DNA vaccines, Protein vaccines, Protein vaccines, Bioinformatics

## Abstract

*Streptococcus agalactiae* is a causative agent of streptococcosis disease in various fish species, including Nile tilapia (*Oreochromis niloticus* Linn.). Vaccination is an effective disease prevention and control method, but limitations remain for protecting against catastrophic mortality of fish infected with different strains of streptococci. Immunoproteomics analysis of *S. agalactiae* was used to identify antigenic proteins and construct a chimeric multiepitope vaccine. Epitopes from five antigenic proteins were shuffled in five helices of a flavodoxin backbone, and *in silico* analysis predicted a suitable RNA and protein structure for protein expression. 45F2 and 42E2 were identified as the best candidates for a chimeric multiepitope vaccine. Recombinant plasmids were constructed to produce a recombinant protein vaccine and DNA vaccine system. Overexpressed proteins were determined to be 30 kDa and 25 kDa in the *E. coli* and TK1 systems, respectively. The efficacy of the chimeric multiepitope construct as a recombinant protein vaccine and DNA vaccine was evaluated in Nile tilapia, followed by *S. agalactiae* challenge at 1 × 10^7^ CFU/mL. Relative percentage survival (RPS) and cumulative mortality were recorded at approximately 57–76% and 17–30%, respectively. These chimeric multiepitope vaccines should be applied in streptococcosis disease control and developed into a multivalent vaccine to control multiple diseases.

## Introduction

Tilapia is a globally economically important aquaculture fish species, particularly in tropical and subtropical countries, such as China and Thailand^1^. Highly intensive farming systems can increase stress and disease outbreaks in cultured fish^2^. Bacterial infectious diseases, such as streptococcosis disease caused by the gram-positive pathogen *Streptococcus agalactiae*, have severe and devastating effects in tilapia aquaculture^3^. Infected fish can develop various symptoms, including an anorexia phase, hyperemic gills, dermal hemorrhages, dark skin pigment, eye lesions, spinal curvature, erratic swimming, and diffuse epithelial tissue proliferation symptoms^3,4^.

*S. agalactiae* is categorized into 11 serotypes: serotypes I-XI^5^. Serotypes Ia, Ib, and III are the most commonly found serotypes in infected fish^6,7^. Serotypes Ia and III have been commonly isolated in Thailand^7^. Vaccines have been used to control disease emergence, including whole-cell inactivated vaccines^8,9^, live attenuated vaccines^10^, recombinant vaccines^11,12^, and DNA vaccines^13,14^. Although whole-cell vaccines exhibit excellent protection in tilapia, they have limitations in controlling heterologous *S. agalactiae* serotypes. Subunit vaccines can surpass this limitation using common antigens present in all serotypes, such as ornithine carbamoyl transferase (OCT), pilus island (PI)-1 ancillary protein 1^15^, CAMP factor, R5 protein, enolase, hemolysin (cyLE)^16^, fibrinogen-binding protein A (FbsA)^17^, and surface immunogenic protein (Sip)^14^.

Multivalent and multiepitope vaccines combining at least three segments or epitopes conjugated by linkers have been presented as alternative disease prevention and control strategies^18^. Various bioinformatics approaches, such as immunoinformatics, molecular dynamics simulation, and protein-protein interaction studies, have been applied to design appropriate and effective multivalent and multiepitope subunit vaccines^19^. Each individual epitope in a chimeric polypeptide vaccine may provide a high efficacy vaccine by inducing and enhancing robust and specific humoral responses in addition to other cellular responses, particularly opsonization activity^20^. Moreover, proper linkers have been considered to minimize steric hindrance of each chimeric epitope and enhance epitope presentation to the host immune system^21^.

Chimeric multiepitope vaccines were generated by combining five different segments of antigenic genes of *S. agalactiae* on the surface of flavodoxin. Two best chimeric multiepitope vaccines were created by molecular modeling analysis and were produced as a recombinant protein vaccine and a DNA vaccine that were shown to effectively protect against streptococcosis disease in tilapia with different immune response patterns. This platform will elucidate the development of vaccines that combine multiple epitopes from different pathogens to create multivalent vaccines that effectively control fish diseases by single vaccination.

## Results

### Immunogenic protein characterization

**Figure 1:**
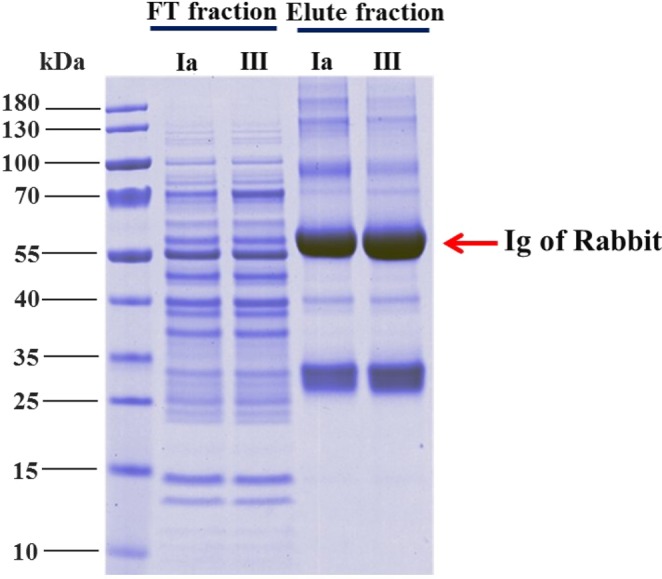
Analysis of the immunogenic protein profile of *S. agalactiae* with a 4–20% gradient NuPAGE gel. The flow-through (FT) fraction represents the unbound *S. agalactiae* proteins, and the eluted fraction represents the immunogenic proteins of *S. agalactiae*. Markedly different protein patterns between the flow-through fraction and eluted fraction were revealed.

Proteins bound to a *S. agalactiae* antibody were eluted from protein A agarose and divided into two fractions. The first fraction was subjected to 4–20% gradient SDS-PAGE to observe the protein features and compare the protein profile from serotypes Ia and III. The second fraction was subjected to LC-MS/MS mass spectrometry to identify the immunogenic proteins. The protein profile from the immunoprecipitation on 1D-SDS-PAGE demonstrated that the major protein (approximately 55 kDa) corresponded to rabbit immunoglobulin. However, several bacterial proteins could not be bound to rabbit immunoglobulin and were removed through the flow-through fraction (FT), whereas the protein that specifically bound to the anti-*S. agalactiae* antibody could be detected in the eluted fraction (Fig. 1).

**Figure 2:**
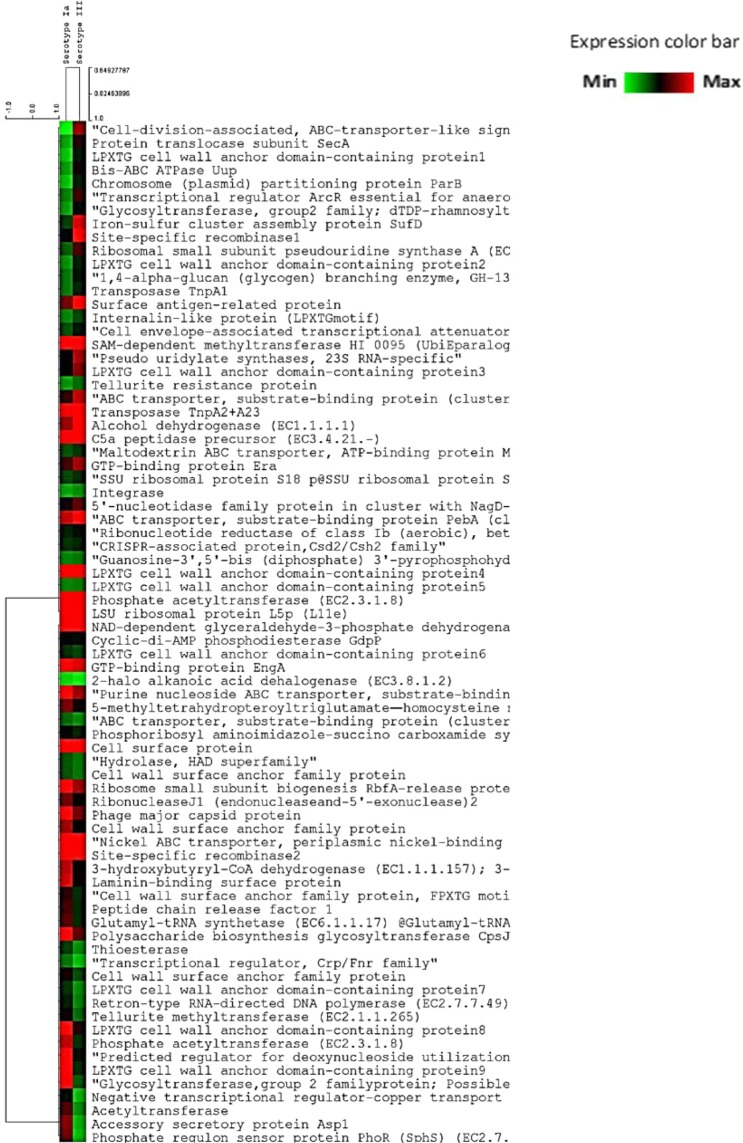
Heat map with hierarchical clustering (HCL) of normalized protein abundance reveals the 79 differentially expressed immunogenic proteins. The expression value showed in the relative intensities ranges from the highest protein abundance (red) to the lowest protein abundance (green) expression value.

Comparative immunoproteomics analysis of *S. agalactiae* serotypes Ia and III was determined by LC-MS/MS and assessed by a Venn diagram (Supplementary Fig. 1). One hundred proteins were matched and identified between serotype Ia and serotype III via in-house protein databases, resulting in 79 shared proteins between serotype Ia and serotype III. The protein expression levels of the 79 common proteins were determined by hierarchical clustering (HCL). Two groups of immunogenic proteins were demonstrated based on their abundance, and 37 proteins were overexpressed in serotype III, whereas there was a lower abundance of 39 immunogenic proteins in serotype III than in serotype Ia (Fig. 2). Regarding specific antigen-antibody interactions, 10 and 11 proteins were uniquely identified in serotypes Ia and III, respectively (Supplementary Figs. 1, 2).

### Linear β-cell epitope prediction and chimeric vaccine design

The epitopes of immunogenic proteins were predicted by the BCPREDS server based on B cell epitopes to be used in chimeric multiepitope vaccine construction. In this study, not only immunogenic proteins from the immunoproteomics analysis were used but also other subunit vaccine candidates were subjected to epitope prediction and combined to produce a chimeric multiepitope vaccine. The amino acid sequences of the C*-β* protein (*BAC*), surface protein rib (*Rib*), LPXTG cell wall anchor domain-containing protein (*SPB1*), surface immunogenic protein (*Sip*), and cell surface protein (*CSF*) showed a different number of epitopes with a BCPredScore of 1.0 (Table 1). However, to construct a chimeric multiepitope vaccine, all epitopes were randomly shuffled on 6 linkers. A total of 7,200 chimeric multiepitope vaccine models were designed.

**Table 1:** Predicted B-cell epitopes from immunogenic proteins used in this study.

Gene	Symbol	Position from N-terminus	Sequence Epitope	BCPred Score	Protein Name	Accession Number
*BAC* gene	BAC1	905	PKTPEAPKIPEPPKTPDVPK	1	C-*β* protein	BAE45252
	BAC2	58	SMAQTDQGNNSSSSELETTK	1		
	BAC3	868	SPKTPEAPHVPESPKAPEAP	1		
	BAC4	841	APDTPQAPDTPHVPESPKAP	1		
	BAC5	820	KGLETNTPETPDTPKIPELP	1		
	BAC6	262	DQEIQEHVKKETSSEENTQK	1		
*Rib* gene	Rib1	745	TPVDTATPGDKPAKVVVTYP	1	Surface protein rib	EAO72273
*Sip* gene	Sip1	296	AQKAPTATPVAQPASTTNAV	1	Surface immunogenic protein	AUP09114
	Sip2	264	PEHVSAPAVPVTTTSTATDS	1		
*CFS* gene	CSF1	68	TVSDLFSDGNNNSSSSKTES	1	Cell surface protein	AIK73093
*SPB1* gene	SPB1_1	398	ATEYTTGADGIITITGLKEG	1	LPXTG cell wall anchor domain-containing protein	WP_000913277

### Molecular modeling of a chimeric multiepitope vaccine

Protein conformation is important for chimeric multiepitope design regarding whether suitable folding can display proper epitopes and maintain high stability. Therefore, a structural domain with an α/β conformation that produces α-helices and parallel β-strands alternatively throughout the backbone likely provides potential bioactivity^22^. Considering α/β fold structure, flavodoxin from *Escherichia coli* [PDB accession code: 3CHY]^23^ was utilized as a linker to combine the epitope fragments from five antigenic proteins.

**Figure 3:**
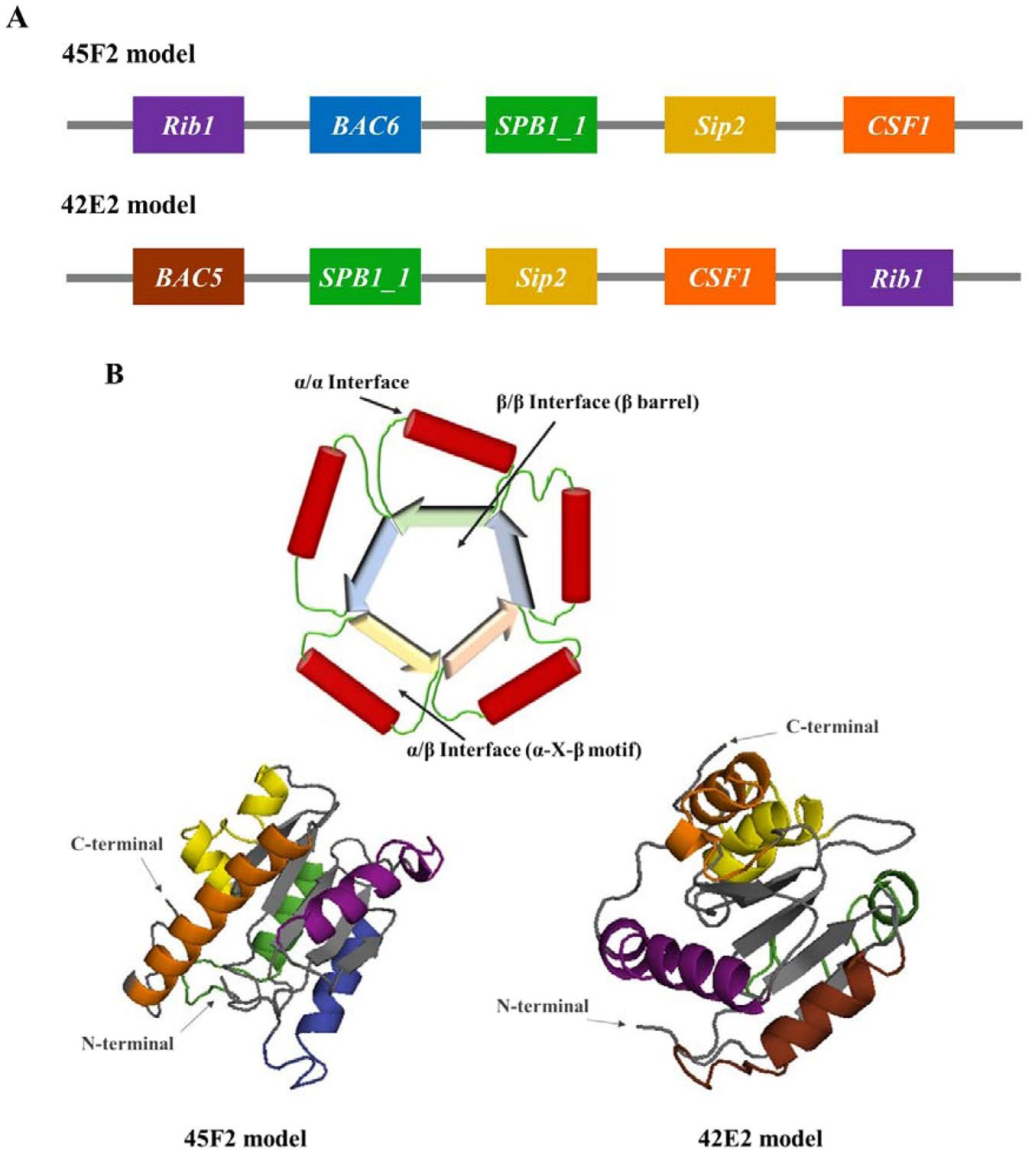
Schematic diagram and predicted 3D model of designed chimeric multiepitope vaccines. (**A**) The schematic diagram depicting the chimeric multiepitope vaccine of the 45F2 and 42E2 constructs containing 5 different B cell epitopes in α-helical structures (*Rip1*, *BAC6*, *BAC5*, *SPB1_1*, *Sip2*, and *CSF1* illustrated in violet, blue, brown, green, yellow and orange, respectively) linked with 6 fragments of a β-pleated sheet of the flavodoxin backbone (gray color). (**B**) The predicted 3D protein structures of the 45F2 and 42E2 models, which were the two best vaccine candidates, are shown as α/β proteins with a flavodoxin fold. Their colors are represented as colors in the schematic diagram, and their N terminus and C terminus are indicated by arrows. These vaccines were designed based on the desirable construction of the TIM-barrel structure, as shown in Fig. 4B.

Predicted epitopes were randomly displayed on the α-helix structure of flavodoxin, generating 1,440 designed models due to the variance of 6 epitopes of BAC and of 2 epitopes of Sip protein. After joining, protein conformation was examined by molecular modeling with 7,200 constructs. I-TASSER and stereochemical qualitative allowance manifested from 45F2 and 42E2 showed appropriate potential tertiary structure with optimal C-scores between −5 and 2. 45F2 and 42E2 also demonstrated the highest score of the amino acid allowance region in the Ramachandran plot. The 45F2 multiepitope model represented 90.2%, 8.5%, and 0.7% of residues located in the most favored, allowed, and disallowed regions, respectively. Meanwhile, the Ramachandran plot regions for the 42E2 designed model comprised 83.0%, 11.6%, and 0.7%, respectively, of the residues (Supplementary Fig. 3). The epitope arrangements in 45F2 and 42E2 were represented in a 3D structure of chimeric proteins, showing that all chosen epitopes were exposed to the protein surface. The five epitopes were displayed as α-helical layers surrounding 3CHY linkers, which appeared as five-stranded parallel β-sheets at the structure’s center, with the order 21345 (Fig. 3).

### Codon optimization of chimeric multiepitope vaccines and plasmid construction

The ectopic expression of bacterial protein in the fish cells may not be achieved due to different codon utilization in the bacterial system. Subsequently, codon optimization of the chimeric multiepitope vaccine was analyzed by GeneArt™’s gene optimization according to ISO 9001 standards (registration no. 1210024212) to apply the codon bias of *Oreochromis niloticus*. The region of an ideal GC content range—between 30% to 70%—was well optimized. Moreover, negative cis-acting sites included internal TATA-boxes, chi-sites and ribosomal sites; AT-rich or GC-rich sequence stretches; RNA instability motifs; repeat sequences; RNA secondary structures; and splice donor and acceptor sites in higher eukaryotes, which were successfully removed from these chimeric multiepitope DNA vaccine sequences. The best two predicted chimeric multiepitope vaccines were designated 45F2 and 42E2.

Codon adaptation index (CAI) presented 45F2 and 42E2 scores that matched in codon utilization with that of Nile tilapia of 0.92 and 0.93, respectively. The codon quality distribution index of 45F2 and 42E2 demonstrated that the codons within the DNA sequence were distributed frequently in 90–100 positions at 77% and 78% (Supplementary Fig. 4A–D). The average GC content of both chimeric multiepitope vaccines was 56% (Supplementary Fig. 4E,F). Single-stranded RNA-folding prediction revealed the minimum free energy (MFE) secondary structure of 45F2 and 42E2 (Supplementary Fig. 5), together with the free energy of the thermodynamic ensemble at -194.44 kcal/mol and −181.16 kcal/mol, respectively.

*N-* and *O-*linked glycosylation sites of 45F2 showed *N-*glycosylation at N^161^ (in the CSF protein fragment), whereas 42E2 showed glycosylation at N^129^ (in the CSF protein fragment). Meanwhile, there were eight (O^122^, O^130^, O^132^, O^133^, O^135^, O^141^, O^169^, and O^171^) and four (O^92^, O^93^, O^95^ and O^150^) putative *O-*glycosylation sites in 45F2 and 42E2, respectively.

The ProtParam server demonstrated a theoretical pI of 4.1 and a molecular mass of 20 kDa for 45F2 and 42E2. The total number of negatively (Asp and Glu) and positively (Arg and Lys) charged amino acid residues of 45F2 was 33 and 13 residues, while for 42E2, there were 31 and 12 residues, respectively.

The estimated half-life of both chimeric multiepitope constructs was approximately 30 h in mammalian reticulocytes (*in vitro*), more than 20 h in yeast (*in vivo*), and over 10 h in *E. coli* (*in vitro*). 45F2 showed aliphatic index and grand average of hydropathicity values of 65.32 and −0.401, respectively, whereas 42E2 showed values of 67.93 and −0.296, respectively. The 45F2 and 42E2 proteins were indicated to be stable proteins, as represented by instability indexes of 31.31 and 25.53, respectively.

**Figure 4:**
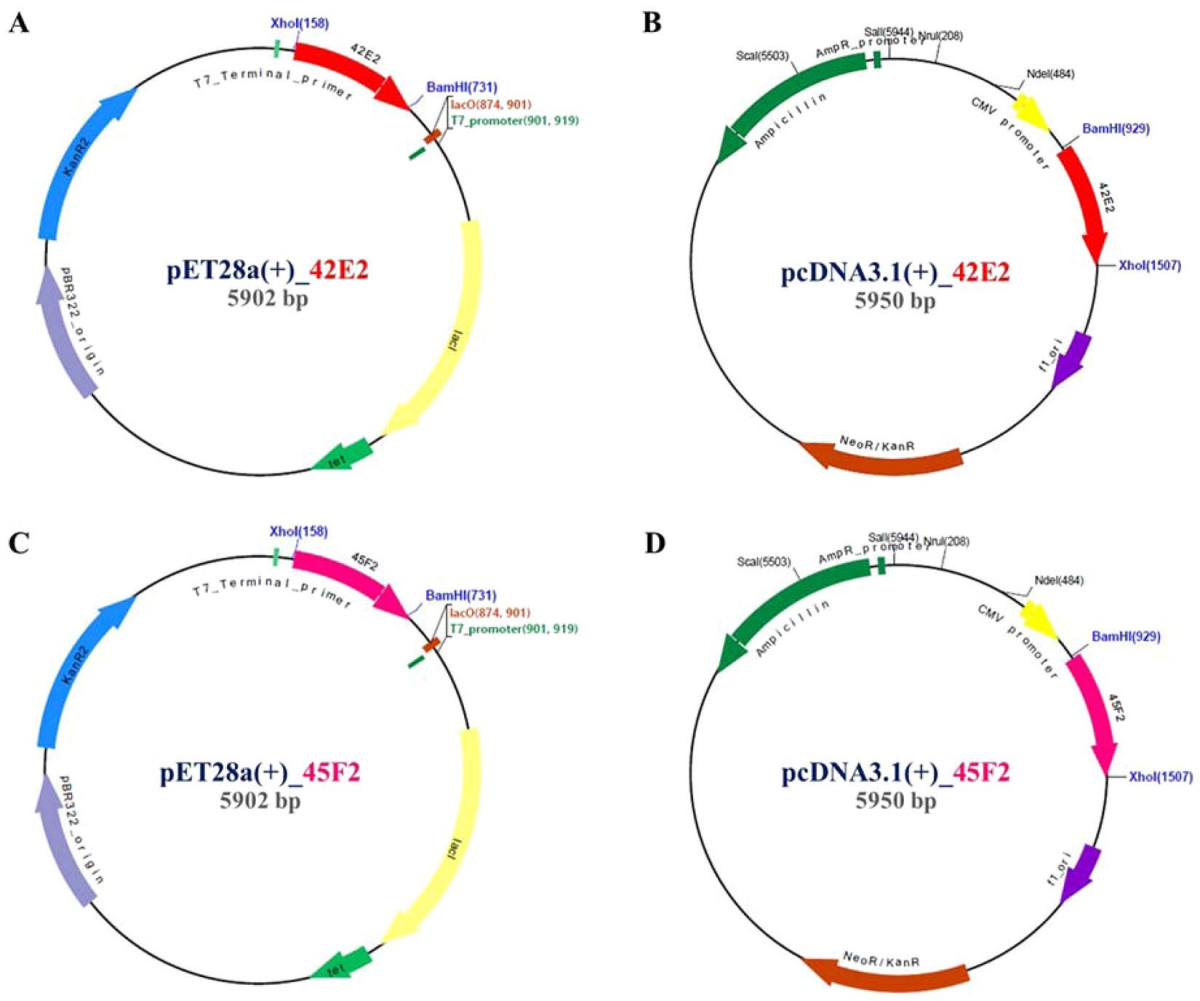
Four plasmid constructs of chimeric multiepitope vaccines. (**A,C**) are the expression plasmids of 42E2 and 45F2 in pET28a, a prokaryotic bacterial system; (**B,D**) represent expression plasmids based on pcDNA3.1, a eukaryotic tilapia cell culture system.

Antigenicity of the 45F2 and 42E2 chimeric multiepitope vaccines was predicted as 0.7538% and 0.7424% at a 0.4% threshold for the bacterial model, consistent with ANTIGENpro server prediction by representing 0.936 and 0.923, respectively. These results indicate that both vaccine candidates have high potential antigenic properties. Conformational B cell epitopes from the 3D protein structure computed by the DiscoTope server demonstrated 11 B cell epitope residues in both 45F2 and 42E2 at a −3.7 threshold (Table 2)^24^. Interestingly, the number of epitopes was reduced when computed at the −2.5 and −1.0 thresholds, with 45F2 showing 6 and 3 B cell epitope residue regions, respectively, while 42E2 contained only 2 and 1 B cell epitope residue regions, respectively (Table 2). Recombinant plasmids harboring 42E2 and 45F2 were constructed, namely, pET28a (+)_42E2 or _45F2 and pcDNA3.1 (+)_42E2 or _45F2, which were used to determine the recombinant chimeric multiepitope vaccine expression (Fig. 4).

**Table 2:** Predicted conformational B-cell epitopes from 3D structure of designed chimeric multiepitope vaccines using DiscoTope 2.0 server.

Position	Residues	Contact No.	DiscoTope score (−1.0 threshold)	DiscoTope score (−2.5 threshold)	DiscoTope score (−3.7 threshold)
**45F2 model**					
	P	4			−2.633
15	Q	3			−3.581
45	E	6			−3.527
49	K	11			−2.743
53	S	7		−2.246	−2.246
56	S	7	−0.053	−0.053	−0.053
57	E	0	0.238	0.238	0.238
58	E	0	−0.324	−0.324	−0.324
59	N	16			−2.917
60	Q	1		−1.810	−1.810
62	P	6		−2.347	−2.347
76					
**42E2 model**					
14	K	0			−3.101
18	T	2			−3.663
22	E	8			−3.441
25	D	9			−3.374
26	T	0	−0.847	−0.847	−0.847
28	K	7			−2.914
30	P	5		−2.396	−2.396
31	E	17			−2.830
124	D	0			−2.791
125	G	3			−2.887
129	S	0			−3.612

### *In vitro* determination of chimeric multiepitope vaccine expression

**Figure 5:**
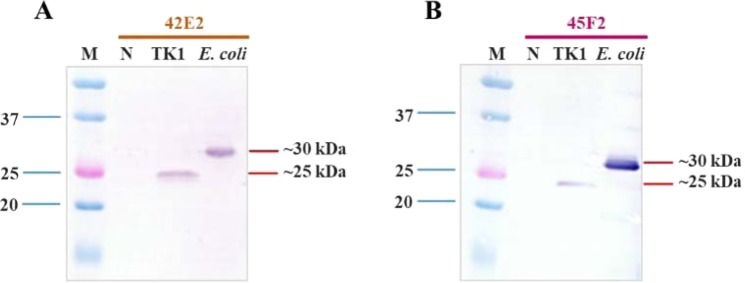
Western blot analysis of chimeric multiepitope vaccine proteins from the bacterial expression system and fish cell (TK1) culture system. (**A**) 42E2 and (**B**) 45F2 proteins expressed in TK1 cells and the *E. coli* Rosetta-gami B (DE3) pLysS strain at approximately 25 kDa and 30 kDa, respectively (M = prestained protein marker; N = noninduced).

Determination of recombinant chimeric multiepitope protein expression was tested in a bacterial expression system and a fish cell (TK-1) culture expression system. These results demonstrated that both chimeric multiepitope proteins could be expressed in both systems, with the expression detectable within 3 h in *E. coli* (30 kDa) and within 7 days post-transfection in TK-1 cells (25 kDa) (Fig. 5). Larger-sized chimeric multiepitope proteins in the *E. coli* expression system resulted from an additional tag at the N-terminus, which was contained in the pET28 expression vector.

### Vaccine efficacy

After vaccination, fish were challenged with *S. agalactiae*, and infected fish showed clinical signs of streptococcosis disease, such as swirling swimming, opaque eye, exophthalmia and abscess. These moribund fish were collected, and bacteria were re-isolated, showing that they were infected with *S. agalactiae* serotype III (Supplementary Fig. S7).

**Figure 6:**
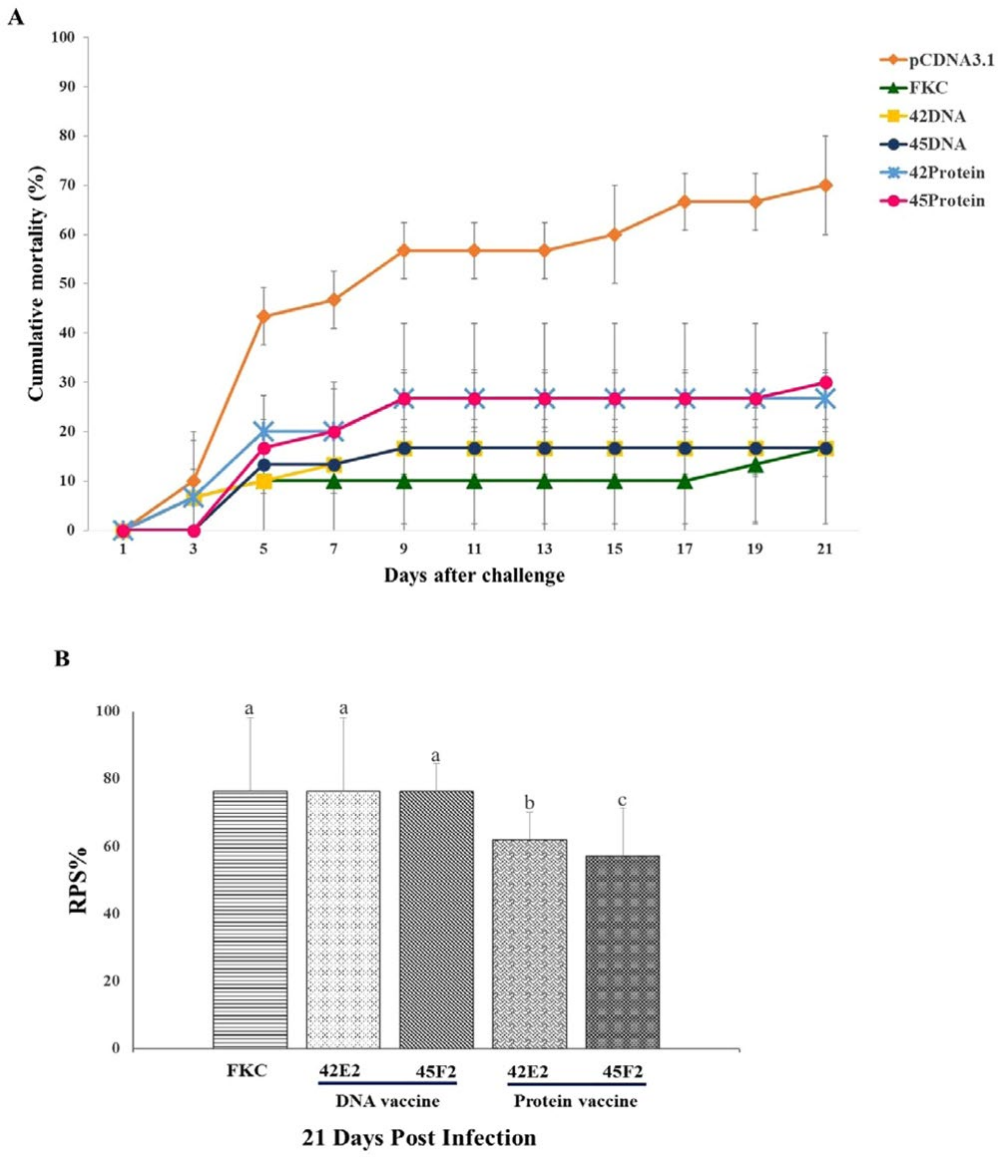
Analysis of chimeric multiepitope vaccine efficacy. (**A**) Cumulative mortality for chimeric multiepitope DNA vaccines and recombinant protein vaccines compared with that for the FKC (positive control) and pcDNA3.1(+) (negative control) groups. (**B**) Relative percentage survival (RPS) of chimeric multiepitope vaccine groups based on that of the control pcDNA3.1 group at 21 days post challenge. Data are represented as the means ± SDs (n = 3). Statistical analysis was performed via a one way ANOVA, compared with the control group (pcDNA3.1). The different letters above the bars indicate significant differences (P < 0.05).

DNA vaccine efficacy testing showed that fish immunized with either 45F2 or 42E2 had cumulative mortality rates of 16.67 ± 5.77% and 16.67 ± 15.27%, respectively, which were not significantly different from those of the FKC-vaccinated fish (*P* > 0.05). However, in the control group [empty vector; pcDNA3.1(+)], 70.00 ± 10.00% mortality was observed at 21 days post-challenge (Fig. 6A).

The recombinant chimeric multiepitope protein vaccination showed that 45F2 and 42E2 produced cumulative mortality rates of 30.00 ± 10.00% and 26.67 ± 5.77%, respectively, which were significantly lower than those of the negative control group, at 70% (*P* < 0.05) (Fig. 6A).

The 45F2 and 42E2 DNA vaccines demonstrated similar patterns of RPS, with 76.19 ± 8.24% and 76.19 ± 21.82%, respectively, which were not significantly different from those of the FKC-immunized fish (76.19 ± 21.82%). However, they were significantly higher than those of the recombinant protein vaccines, which showed 61.90 ± 8.24% and 57.14 ± 14.28% for 42E2 and 45F2, *P* < 0.05, respectively (Fig. 6B).

### Immune response

**Figure 7:**
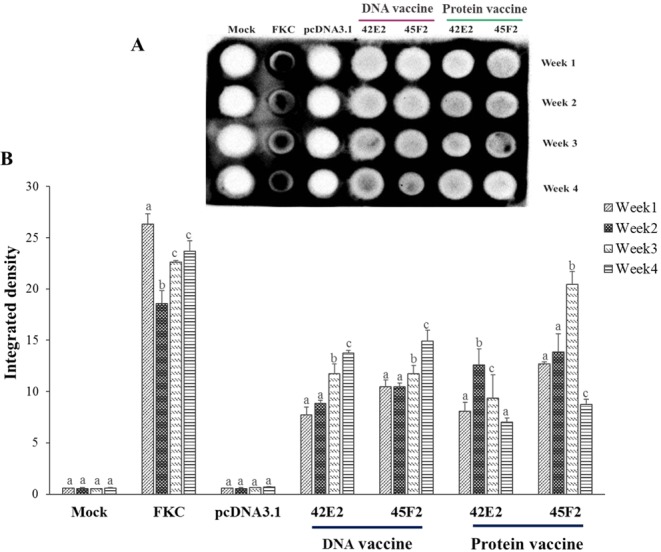
Dot blot analysis of serum from vaccinated fish. (**A**) The detectable antibody from vaccinated fish serum is shown on the membrane compared with that for the mock, FKC (formalin killed cell) and pcDNA3.1 group (negative control) against recombinant protein of multiepitope vaccine themselves of FKC vaccine at 1 month. (**B**) The integrated density from all the dot blot results were converted to the values using the ImageJ 1.x server. Data are represented as the means ± SDs (n = 2). The different letters above the bars indicate significant differences within treatment (P < 0.05).

To determine the immune response, dot blot analysis of serum prepared from 42E2- or 45F2-vaccinated fish was used. It was demonstrated that the DNA vaccine could gradually activate the production of fish antibodies from the 1^st^ to the 4^th^ week. The pattern of antibody response differed from that for the recombinant protein vaccine, with the highest activation of antibody production being significantly produced in the 2^nd^ - 3^rd^ week and suddenly dropping in the 4^th^ week. The highest induction was observed in FKC-immunized fish (Fig. 7). Dot blot analysis of vaccinate fish sera against whole cell lysate of *S. agalactiae* serotype Ia and III demonstrated that fish vaccinated with recombinant protein vaccine 42E2 and 45F2 showed cross-reactivity to whole cell lysate of *S. agalactiae* serotype Ia and III (Supplementary Fig. S8).

## Discussion

For the reverse vaccinology approach, computational analysis using a variety of bioinformatics tools is robust and beneficial when identifying appropriate vaccine candidates^18^. Bacterial genomics and proteomics analysis indeed help researchers analyze proteins, short domains, and pathogenic epitopes that provide high immunogenicity and high antigenicity for multimeric vaccine development^25^. Therefore, immunoproteomics should be applied as a preliminary process to screen antigenic proteins and minimize potential candidates for vaccine development^21^.

Several immunogenic proteins in this study were described previously, such as C5a peptidase and laminin-binding surface protein (lmb), which are cell surface proteins that have an important function in chemoattractant activities and are proteins promoting invasion of group B streptococcus (GBS)^26,27^. However, the current immunoproteomics analysis from this study identified new immunoreactive proteins, such as bacteriocin transport accessory protein, dihydrofolate reductase, SSU ribosomal protein S8p, transposase TnpA, 1,4-alpha-glucan, cell wall surface anchor family protein, and the GTP-binding protein Era. As expected, most of these are cell surface proteins, which are suggested to be associated with bacterial virulence^27,28^. Subsequently, the identified immunoreactive proteins may be used in further vaccine development.

Multiepitope vaccines are an interesting issue since constructed vaccines designed by *in silico* analysis may elicit cellular immunity and provide effective responses^25,29^. It is known that immunodominant B cells could strongly induce both cellular and humoral immunity; thus, evaluation of B cell epitopes was performed to identify potent epitopes before integrating them to produce a multiepitope vaccine. Moreover, this vaccine type is more efficient than whole antigens for controlling *Staphylococcus* spp. infections^20,30^. From the present study, linear B cell epitope prediction was assessed and identified 11 potent epitopes from 5 common immunogenic and virulence proteins that were present in serotypes Ia and III. The previous studies supported that one of the chosen proteins, Sip, represented a highly conserved protein among GBS isolates and showed cross-protective immunity against GBS infections^11,13,14,31^. Prediction of candidate antigenic proteins can be used to select the bacterial strains that carry antigenic genes, as well as to determine high expression levels in the target host and the accessibility of particular antigens in host organisms^16^. Therefore, these selected immunogenic proteins might be suitable for consideration in a rational vaccine design.

Rational chimeric multiepitope vaccine design was achieved by randomly combining epitopes from 5 immunogenic proteins and conjugating with core structures of flavodoxin (*β*-1–5–3CHY) to produce a secondary structure with α/β folding. In addition to the α/β-type folding of flavodoxin, it was also useful to construct our chimeric multiepitope vaccine by forcing the 5 chosen epitope segments to fit within 5 α-helix loops and protrude out of the 3D-folded structure since that configuration benefits protein solubility by exposure to water molecules^23^. Additionally, this linker may promote the solubility of the constructed vaccine and help enhance the recognition of the vaccine by the host’s immune system, which contributes to vaccine efficacy. 45F2 and 42E2 presented the most favored region of protein folding, with the stereochemical quality representing the disallowed region at only 0.7%, which is acceptable since the minimum quality should be less than 2%^32^. It is suggested that *in silico* analysis could design a chimeric multiepitope vaccine that could probably manifest effective properties^33,34^.

To achieve a high level of protein expression in Nile tilapia, codon optimization was conducted to improve the transcription and translation capability by removing all possible cis-acting sequence motifs, which may have a negative impact on protein expression. Both proteins had a CAI > 0.8 and a codon with frequent distribution (CFD) > 30%, which are acceptable for high expression in the target organism^18,21^. The GC content of 45F2 and 42E2 was optimized between 30–70% and had a suitable thermodynamic ensemble free energy, which allowed RNA folding and thermodynamic stability^35,36^. The overall points suggested that the modeled 45F2 chimeric multiepitope vaccine was clearly the best candidate vaccine.

Numerous effective single-serotype GBS vaccines have been reported, including vaccines for controlling streptococcosis in tilapia^10,11,13,14,17,37,38^. However, it is known that single-serotype whole-cell inactivated vaccines have limitations during cross-prevention against different serotypes. For instance, a *S. iniae* vaccine (serotype I) could not protect Atlantic salmon from infection by *S. iniae* (serotype II)^39^. Meanwhile, mixed-serotype vaccines (serotypes IV and VII) could promote antiserum levels and enhance the survival rate of newborn pups against streptococcal infection^40^. Although formalin-killed vaccines generally provide highly protective effects compared with those of subunit vaccines and DNA vaccines, the subunit and DNA vaccines may replace the original formalin-killed vaccines or inactivated vaccines due to their promising efficacy, which are similar to those of inactivated vaccines, and longer shelf life^18,33^. Evidence suggests that DNA and subunit vaccines can efficiently trigger the immune system and promote protective efficacy, with an RPS value greater than 50%^11,13,14,17^. Nevertheless, these vaccines have limitations, such as their mass production costs, and they may require various optimizations to obtain the highest stable storage conditions^18,41^.

Regarding this idea, a chimeric multiepitope vaccine composed of different epitopes from different proteins common in both serotypes Ia and III was generated to achieve broad protection against different serotypes and increase their stability. Interestingly, the designed chimeric multiepitope DNA vaccine and protein vaccine exhibited effective prevention in Nile tilapia against *S. agalactiae*, with efficacy similar to that of the whole-cell inactivated vaccine. This evidence supports the strategy of rational vaccine design through B cell recognition using *in silico* analysis. Importantly, immunoproteomics analysis could assist the preliminary determination of suitable immunogenic proteins for vaccine development due to the distinct antigenic determinants that can mediate dissimilar immune responses. The criterion in immunogenic protein selection for vaccine development has focused on the ability of a particular protein to induce an immune response. Among 79 identified proteins shared in both serotypes, in addition to providing the highest BCPRED scores (Table 1), the 5 proteins chosen were also reported as virulence proteins and used as vaccine candidates for streptococcosis disease prevention^13^. For example, C-*β* protein (*BAC*) can lead to antibody production through Fc region binding of human IgA^42^. Sip protein has been shown to mediate protection against streptococcal infection^11,13,14^. Additionally, the chosen immunogenic protein should be conserved among *Streptococcus* spp., so it would be suitable for application in cross-reactive prevention among *S. agalactiae* serotypes^11,43^. Moreover, it should be mentioned that peptide vaccines or epitopes with only 30 amino acid residues may trigger immune responses through binding directly to MHC-I or MHC-II molecules. These molecules localize to nonprofessional antigen-presenting cells. Vaccines containing proteins with longer amino acid sequences can enhance the presentation of epitopes to dendritic cells due to T cell induction^25,44,45^. Herein, the comparative efficacy of both the 45F2 and 42E2 DNA and recombinant protein vaccines indicated that the DNA vaccine provided a higher efficacy than the recombinant protein vaccine. This result suggests that the DNA vaccine can prolong the activation of the immune response by triggering both humoral and cellular immune responses^46,47^. Moreover, the clearance rate of the recombinant protein vaccine in the host system may be faster than that of the DNA vaccine. This difference implies that the DNA vaccine can enter the host cell to produce chimeric multiepitope protein, with that protein existing in the host system for longer than the recombinant protein vaccine, thus enhancing its bioavailability.

Taken together, these data indicate that the antigen combination has shown promise for streptococcosis disease control in Nile tilapia. This research demonstrated a novel platform for rational vaccine design based on chimeric vaccine development that used flavodoxin with a Tim-barrel structure as a template. Our chimeric protein backbone is suitable for presenting epitopes to be recognized by the host immune system. With 5 epitopes, it could activate antibody production and demonstrated promising protection against bacterial disease similar to that of a whole-cell inactivated vaccine. This platform will promote the production of multivalent vaccines to control multiple diseases and for other applications in the future.

## Materials and Methods

### Experimental fish, bacterial strain and antibody

All male *S. agalactiae-*free Nile tilapia (*Oreochromis niloticus* Linn.) were obtained from a commercial GAP farm in Thailand. The experiments were conducted in accordance with guidelines approved by the National Research Council of Thailand. The experimental fish were anesthetized with clove oil to minimize stress during vaccination and challenge testing. *S. agalactiae* serotypes Ia and III were cultured as described previously^7^. *S. agalactiae* serotype III was used for polyclonal antibody (pAb) production, which was kindly provided by Prof. Ikuo Hirono, TUMSAT, Japan. Antibody against IgM of Nile tilapia was kindly provided by Assist. Prof. Eakapol Wangkahart. Mahasarakham University, Thailand.

### Immunoproteomics analysis

*S. agalactiae* was grown in BHI broth at 30 °C with agitation until reaching exponential phase. Bacterial cells were collected by centrifugation, lysed in 100 µL of lysis buffer [Tris-buffered saline (TBS) with 1% Tween-20 and 0.01% lysozyme] and incubated at 50 °C for 20 min following sonication on ice. Protein A agarose beads (Cell Signaling, USA) were added to the bacterial protein lysate, and nonspecific proteins were removed by 10 min of centrifugation at 10,000 × g at 4 °C. Clarified supernatant was supplemented with 5% glycerol and then with a pAb specific to *S. agalactiae* serotype III (1:500 dilution). Then, 30 µL of protein A agarose beads were added to separate bound immunogenic proteins, and the bound proteins were separated by acetone precipitation [1:5 (v/v)]. Precipitated proteins were solubilized in 20 mM Tris-HCl with 0.5% SDS, and a Lowry assay was used to measure the protein concentration. The protein profile was assessed by fractionating 25 µg of protein on a NuPAGE 4–12% Bis-Tris Protein gel (ThermoFisher, USA).

3 µg of immunogenic protein was mixed with a lysis buffer (0.1% RapidGest SF in 20 mM ammonium bicarbonate) and 5 mM DTT in 10 mM ammonium bicarbonate at 60 °C for 3 h. This step was followed by incubation with 15 mM iodoacetamide (IAA) in 10 mM ammonium bicarbonate at room temperature for 45 min in the dark. The protein solution was cleaned up by a Zeba Spin Desalting Column before digestion with 50 ng of sequencing-grade trypsin (Promega, Germany) at 37 °C for 6 h. Tryptic peptides were dried at 44 °C under a vacuum and then protonated with 0.1% formic acid in LC water before injection into an LC-MS/MS.

The tryptic peptides’ immunoproteomics profiles were analyzed using an Ultimate™ 3000 Nano/Capillary LC System (Dionex, UK) and Hybrid quadrupole Q-Tof impact II™ (Bruker Daltonics GmbH, Germany) equipped with a Nano-CaptiveSpray ion source. First, 500 nL of extracted peptide was subjected to a trapping column (Thermo Scientific, PepMap100, C18, 300 μm i.d. × 5 mm) through a full loop injection before being resolved in an analytical column (PepSwift C18 Nano Column, 100 μm × 15 cm, i.d.) at 60 °C. The linear gradient method was used to elute peptides with mobile phase A (0.1% formic acid in water) and mobile phase B (0.1% formic acid in 80% acetonitrile) at a 0.35 µL/min constant flow rate into the mass spectrometer. Electrospray ionization was conducted at 1.6 kV using CaptiveSpray. Mass spectra (MS) and MS/MS spectra were fully acquired in positive ion mode (Compass 1.9 for otofSeries software, Bruker Daltonics). Mass accuracy was assessed using positive detection mode after internal calibration with sodium trifluoroacetate (Na-TFA) within 1.6 ppm. Raw LC-MS/MS spectra were collected using CompassXport Version 3.0.9.2 (Bruker Daltonics GmbH, Germany) to convert all spectra into the mzXML data format. The mzXML files of the LC-MS/MS datasets for label-free quantification of peptides were evaluated based on the MS profile by Maxquant software.

### Chimeric multiepitope vaccine design

The linear B cell epitope was predicted by BCPRED^48^. The SCOP and CATH databases were used to design an appropriate chimeric multiepitope vaccine structure^49^ using *BAC* (accession no. [AN]: BAE45252), *Rip* (AN: EAO72273), *Sip* (AN: AUP09114), *CFS* (AN: AIK73093), and *SPB1* (AN: WP_000913277). A 3D structure was rendered by I-TASSER (Iterative Threading ASSEmbly Refinement) using the qualifying C-score value as a confidence score^32^. To refine the tertiary structure, the derived I-TASSER results in the PDB files were prepared using the GalaxyRefine server, which performed a repeated structure perturbation, and the best structural relaxation candidates were chosen^19^. Moreover, to obtain the best chimeric multiepitope vaccine candidates, the residues were determined according to residue stereochemical quality for all the refined chimeric multiepitope models and validated by the PROCHECK program v.3.5.4 to generate Ramachandran plots^50^.

### Codon optimization

Amino acid sequences were reverse-translated to nucleotide sequences using Nile tilapia codon usage (*Oreochromis niloticus* [gbvrt]: 113). The codon adaptation index (CAI) of the designed vaccine candidates’ nucleotides was analyzed by an optimizer program (http://genomes.urv.es/OPTIMIZER/) and combined with GeneArt^TM^’s gene optimization process (Thermo Fisher Scientific, USA). The secondary structure of the single-stranded RNA folding and free energy of the thermodynamic ensemble were calculated by the RNAfold web server^51^. The optimized DNA sequence was synthesized by GeneArt^®^ Gene Synthesis (Thermo Fisher Scientific, USA)^52^.

### Chimeric multiepitope vaccine characterization

*N-*linked and *O-*linked glycosylation sites were evaluated using NetNGlyc 1.0 Server^53^ and NetOGlyc 4.0 Server^54^. The theoretical pI (isoelectric point), MW (molecular weight), composition of positive and negative residues, estimated half-life, extinction coefficient, aliphatic index, and grand average of hydropathicity (GRAVY) were revealed using the ProtParam server of ExPASy^55^. Antigenicity was analyzed with the VaxiJen v2.0 server^56^ and ANTIGENpro software^57^. The DiscoTope 2.0 server was employed to define discontinuous B cell epitopes at the default threshold of −1.0 to −3.7^24^.

### Chimeric multiepitope vaccine preparation

The DNA vaccine and recombinant vaccine expression vector were constructed by inserting the synthesized nucleotides of the 45F2 and 42E2 genes into the pET28a (+) and pcDNA3.1 (+) vectors at the *BamH*I and *Xho*I sites. For prokaryotic expression, the pET28a vectors harboring chimeric multiepitope vaccines—45F2 or 42E2—were transformed into *E. coli* Rosetta-gami (DE3) pLysS strains (Novagen), and protein expression was induced at 30 °C for 3 h with 0.1 mM IPTG.

To verify the ectopic expression of the chimeric multiepitope DNA vaccine, pcDNA3.1(+)_42E2 or _45F2 was transfected into TK1 (Tilapia Kidney 1) tilapia cells using Effectene Transfection Reagent (QIAGEN, Germany). The transfected fish cell cultures were maintained with Leibovitz’s L-15 media containing 10% FBS and penicillin-streptomycin at 25 °C, and DNA vaccine expression was determined after 1 week.

Recombinant chimeric multiepitope protein was purified by Ni-NTA agarose beads (Qiagen) with a gradient concentration buffer of imidazole ranging from 5 mM to 500 mM. Subsequently, the gel filtration chromatography method was performed by fast protein liquid chromatography (FPLC) incorporated with a HiPrep 16/60&26/60 Sephacryl S-300 High-Resolution column (GE Healthcare, USA) using a 1 × PBS buffer with a 1 mL/min flow rate. Recombinant protein detection was confirmed by SDS-PAGE analysis and Western blot analysis using an anti-His tag antibody (recombinant protein vaccine) or an anti-flag (rabbit IgG) (DNA vaccine) and anti-rabbit antibody conjugated to AP (alkaline phosphatase).

### Vaccine efficacy analysis

To evaluate vaccine performance, Nile tilapia (*O. niloticus*) were immunized with chimeric multiepitope vaccines (recombinant protein and DNA vaccines), followed by bacterial challenge. A total of 6 experimental groups, namely, 1) the 45F2 recombinant protein vaccine, 2) 42E2 recombinant protein vaccine, 3) 45F2 DNA vaccine, 4) 42E2 DNA vaccine, 5) formalin-killed (FKC) *S. agalactiae* vaccine^58^, and 6) pcDNA3.1(+) [empty vector], were conducted in triplicate. Before vaccination, 25 streptococcosis-free Nile tilapia (60 ± 5 g) were transferred into 18 glass aquarium tanks containing 30 L of water for one week. After a week of acclimatization, fish were vaccinated according to above mentioned groups. All fish were maintained under running and aerated water at 30 ± 3 °C and fed with commercial pellet feed twice a day.

For the chimeric multiepitope protein vaccination, purified 45F2 and 42E2 proteins were mixed with Montanide ISA 763 (Seppic, France) in a 7:3 ratio prior to intraperitoneal injection with 200 µg of protein per fish. For the chimeric multiepitope DNA vaccine, plasmid DNA of 45F2 and 42E2 were purified by ultracentrifugation using a CsCl gradient^59^ and dissolved in TE buffer (pH 8.0) to obtain a concentration of 0.1 µg/µL. The DNA vaccine was applied to the fish with 10 µg of DNA through intramuscular injection. FKC and pcDNA3.1(+) were used as positive and negative controls, respectively. The schedule of vaccine efficacy analysis and immune response analysis was demonstrated in Supplementary Fig. 9.

For the immune response analysis, blood was drawn from the caudal vein to separate serum for the immunoblotting assay, and those fish were transferred to another separate tank. The analysis was performed every week, using 3 fish in each treatment from the 1^st^ week to the 4^th^ week.

After one month of vaccination, 10 vaccinated fish in each treatment group were taken from among the remaining fish for serum collection and anesthetized with eugenol before challenge with *S. agalactiae* (serotype III) at 1 × 10^7^ CFU/mL through IP administration. Mortality and clinical signs of infected tilapia were recorded daily for 3 weeks. The brain, head kidney, and liver were collected from moribund fish for bacterial isolation and identification^7^. Cumulative mortality and relative percentage survival (RPS) were calculated^60^. A one way analysis of variance (ANOVA) was used for statistical analysis and P < 0.05 was considered significant.

### Dot-blot immunoassay

To detect the antibody response after immunization, antibody production was evaluated through dot blot analysis using the Minifold^®^ I dot blot system (GE Healthcare, Germany). Briefly, 20 µL of purified 42E2, 45F2 proteins, or a whole-cell lysate of *S. agalactiae* (10 µg/mL) were spotted on a nitrocellulose membrane and blocked with blocking solution (0.1% BSA in TBST) before adding 10 µL of serum of the different treatment groups as above mentioned. Then, the membrane was probed with a primary antibody (anti-IgM at 1:5,000) for 1.5 h, followed by washing 3 times with TBST buffer and 45 min of incubation with an anti-mouse IgG HRP-linked Ab (1:10,000). Subsequently, the signal was detected with a ChemiDoc™ Imaging System (Bio-Rad) after adding a substrate reagent (PerkinElmer, USA). The integrated density of the dot blot was analyzed by ImageJ (version 1.x)^61^.

## Supplementary information

Supplementary information 
