# Supplementary material for: Novel Chimeric Multiepitope Vaccine for Streptococcosis Disease in Nile Tilapia (Oreochromis niloticus Linn.)
Source: Sci Rep. 2020 Jan 17;10:603. doi: 10.1038/s41598-019-57283-0 (PMC6969146; doi:10.1038/s41598-019-57283-0)

Novel Chimeric-multiepitope Vaccine for Streptococcosis Disease in Nile Tilapia (*Oreochromis niloticus*, Linn.)

Ansaya Pumchan[1], Sucheewin Krobthong[2], Sittiruk Roytrakul[2], Orathai Sawatdichaikul[3],

Hidehiro Kondo[4], Ikuo Hirono[4], Nontawith Areechon[5], Sasimanas Unajak[1,6,7*]

Supplementary information

S1: Venn-diagram representing immunogenic protein identified by LC-MS/MS. The number in diagram represent immunogenic proteins uniquely identified in each serotype, and the number in the corresponding overlapping areas represent of differentially expressed immunogenic proteins that are sharing in both serotypes that given.

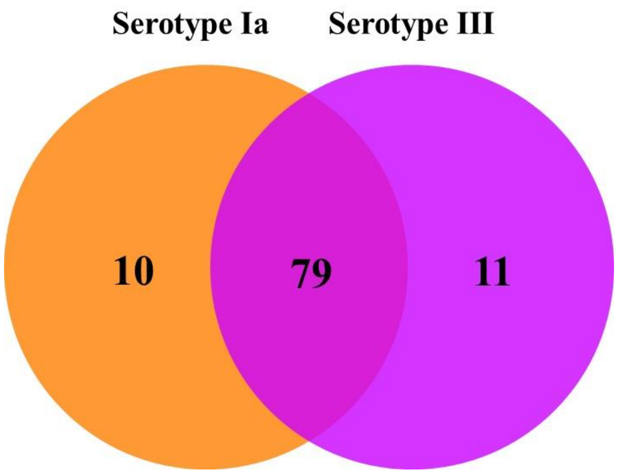

S2: List of immunogenic proteins that found in both serotype Ia and III and the unique immunogenic proteins.

Immunogenic protein name	Peptides [No.]	MW [kDa]	Relative intensity	
			Serotype Ia	Serotype III
Cell-division-associated, ABC-transporter-like signaling protein FtsE	5	26.094	11.20762	17.19422
2-halo alkanoic acid dehalogenase (EC3.8.1.2)	3	34.945	12.03435	11.62872
LPXTG cell wall anchor domain-containing protein_1	3	29.623	12.38775	15.57483
Chromosome (plasmid) partitioning protein ParB	3	29.237	12.50695	15.45677
Protein translocase subunit SecA	4	95.312	12.90032	16.45034
Integrase	4	44.586	13.07433	13.50407
Bis-ABC ATPase Uup	4	71.088	13.11387	16.32592
Glycosyltransferase, group2 family; dTDP-rhamnosyltransferase RfbF (EC2.-.-.-)	3	32.954	13.22405	15.86361
Tellurite resistance protein	3	46.19	13.37205	14.07062
LPXTG cell wall anchor domain-containing protein_2	3	21.008	13.39178	15.47757
LPXTG cell wall anchor domain-containing protein_3	3	20.367	13.57648	13.76911
Guanosine-3',5'-bis (diphosphate) 3'-pyrophosphohydrolase (EC3.1.7.2) /GTP pyrophosphokinase (EC2.7.6.5), (p)ppGpp synthetase II	4	84.207	13.62297	13.89159
Internalin-like protein (LPXTGmotif)	4	160.49	13.64149	14.71644
Transcriptional regulator ArcR essential for anaerobic expression of the ADI pathway, Crp/Fnr family	4	26.766	13.64442	16.57865
Transposase TnpA_1	3	29.581	13.84539	15.34976

Transcriptional regulator, Crp/Fnr family	3	25.735	13.86157	12.5886
1,4-alpha-glucan (glycogen) branching enzyme, GH-13-type (EC2.4.1.18)	3	73.341	13.87546	15.70852
ABC transporter, substrate-binding protein (cluster3,basicaa/glutamine/opines) /ABC transporter, permease protein (cluster3,basicaa/glutamine/opines)	4	57.132	14.19499	13.63719
Ribosomal small subunit pseudouridine synthase A (EC5.4.99.19)	4	27.095	14.37518	16.66025
Cell wall surface anchor family protein_1	3	52.934	14.43365	14.53473
Hydrolase, HAD superfamily	3	51.776	14.43495	13.84343
Cell wall surface anchor family protein_2	4	56.357	14.46289	13.86041
Cell envelope-associated transcriptional attenuator LytR-CpsA-Psr, subfamily F1	3	52.55	14.52203	15.5966
Maltodextrin ABC transporter, ATP-binding protein MsmX	4	42.094	14.57359	15.07707
SSU ribosomal protein S18 p@SSU ribosomal protein S18p, zinc-independent	3	9.2196	14.72345	15.22129
Thioesterase	3	14.417	14.78361	13.47459
Iron-sulfur cluster assembly protein SufD	4	46.506	14.83185	17.53359
LPXTG cell wall anchor domain-containing protein_4	7	59.946	15.09816	13.536
CRISPR-associated protein,Csd2/Csh2 family	3	31.849	15.13121	15.43476
LPXTG cell wall anchor domain-containing protein_5	4	99.573	15.19825	14.82038
Retron-type RNA-directed DNA polymerase (EC2.7.7.49)	4	49.297	15.49713	13.7195
Ribonucleotide reductase of class Ib (aerobic), betasubunit (EC1.17.4.1)	4	36.919	15.50925	15.88617
Negative transcriptional regulator-copper transport operon	4	15.748	15.77213	12.78326
Site-specific recombinase	3	61.009	16.00494	18.6631
Phosphoribosyl aminoimidazole-succino carboxamide synthase (EC6.3.2.6)	3	26.982	16.05059	15.40015
Cyclic-di-AMP phosphodiesterase GdpP	5	73.978	16.0955	15.91868

LPXTG cell wall anchor domain-containing protein_6	3	48.939	16.09583	16.98348
Pseudo uridylate synthases, 23S RNA-specific	3	37.598	16.21176	17.22091
Tellurite methyltransferase (EC2.1.1.265)	3	33.042	16.28452	14.19098
5'-nucleotidase family protein in cluster with NagD-like phosphatase	3	50.68	16.32917	16.80133
Cell surface protein_1	6	118.03	16.33858	17.08701
Cell wall surface anchor family protein_3	3	54.362	16.34676	14.6557
Cell wall surface anchor family protein, FPXTG motif_1	7	100.95	16.43554	15.24911
ABC transporter, substrate-binding protein (cluster3,basicaa/glutamine/opines)	3	28.947	16.5323	17.37382
Peptide chain release factor 1	3	40.598	16.59393	15.38637
GTP-binding protein Era	3	34.206	16.61137	17.17602
Acetyltransferase	3	21.044	16.69493	13.44773
Glutamyl-tRNA synthetase (EC6.1.1.17) @Glutamyl-tRNA (Gln) synthetase (EC6.1.1.24)	3	55.555	16.72401	15.30317
RibonucleaseJ1 (endonucleaseand-5'-exonuclease)	4	60.925	16.89451	16.04778
Phosphate regulon sensor protein PhoR (SphS) (EC2.7.13.3)	3	65.533	16.92555	12.53304
5-methyltetrahydropteroyltriglutamate—homocysteine methyltransferase (EC2.1.1.14)	3	84.66	16.95697	16.30217
Surface antigen-related protein	4	60.155	16.96229	18.65868
Accessory secretory protein Asp1	4	60.428	17.04025	12.78060
Alcohol dehydrogenase (EC1.1.1.1)	3	35.443	17.17543	17.89853
Cell wall surface anchor family protein_4	4	54.36	17.18621	16.24068
Cell wall surface anchor family protein, FPXTG motif_2	3	100.95	17.23491	16.20241
3-hydroxybutyryl-CoA dehydrogenase (EC1.1.1.157); 3-hydroxyacyl-CoA dehydrogenase (EC1.1.1.35)	3	35.459	17.34343	16.21309

ABC transporter, substrate-binding protein PebA (cluster3,basicaa/glutamine/opines)	4	30.522	17.40041	17.88626
Phosphate acetyltransferase (EC2.3.1.8)	3	35.877	17.40831	15.14629
Laminin-binding surface protein	3	34.168	17.46348	16.24685
Transposase TnpA_2	3	22.289	17.70144	18.50742
Glycosyltransferase,group 2 familyprotein; Possible alpha-rhamnosyltransferase, RhaT (EC2.4.1.-)	3	35.925	17.74255	14.40421
Purine nucleoside ABC transporter, substrate-binding protein	7	36.128	17.78040	17.18069
Phage major capsid protein	3	45.421	17.84083	16.88929
LPXTG cell wall anchor domain-containing protein_7	4	33.189	17.87454	15.23586
Ribosome small subunit biogenesis RbfA-release protein RsgA	3	33.422	17.93243	17.15896
GTP-binding protein EngA	3	48.984	18.14808	17.59397
Predicted regulator for deoxynucleoside utilization, GntR family	3	27.811	18.40175	15.73307
Polysaccharide biosynthesis glycosyltransferase CpsJ	4	37.256	18.41159	16.82625
LPXTG cell wall anchor domain-containing protein_8	4	15.812	18.86006	16.43249
C5a peptidase precursor (EC3.4.21.-)	6	86.929	19.22783	19.97421
Phosphate acetyltransferase (EC2.3.1.8)	5	35.877	19.30624	19.56037
SAM-dependent methyltransferase HI 0095 (UbiEparalog)	4	28.304	19.39402	20.60187
Nickel ABC transporter, periplasmic nickel-binding protein NikA (TC3.A.1.5.3)	3	61.536	19.98954	18.75532
Site-specific recombinase	4	62.865	20.0446	18.7713
Cell surface protein_2	12	118.03	20.32608	19.49348
NAD-dependent glyceraldehyde-3-phosphate dehydrogenase (EC1.2.1.12)	13	36.005	21.11299	20.96719
LPXTG cell wall anchor domain-containing protein_9	3	21.983	21.30341	21.70696

LSU ribosomal protein L5p (L11e)	4	19.814	21.69982	21.76065
Putative oxidoreductase	3	48.004	-	12.36791
Efflux ABC transporter, ATP-binding protein	4	26.330	-	13.83269
Bacteriocin transport accessory protein, putative	3	14.068	-	14.08647
Alpha-L-Rhaalpha-1,3-L-rhamnosyltransferase (EC2.4.1.-)	3	36.784	-	14.76570
Ribose ABC transport system, periplasmicribose-binding protein RbsB (TC3.A.1.2.1)	3	34.053	-	14.81633
Cell wall surface anchor family protein_5	3	56.387	-	14.98611
Ribonuclease J1 (endonucleaseand-5'-exonuclease)	3	60.926	-	15.41396
Hypotheticalprotein_1	3	43.766	-	15.69794
5-formyltetrahydrofolate cyclo-ligase (EC6.3.3.2)	3	20.166	-	16.22530
Dihydrofolate reductase (EC1.5.1.3)	4	18.943	-	17.87855
SSU ribosomal protein S8 p (S15Ae)	3	14.785	-	18.22717
Asparaginyl-tRNA synthetase (EC6.1.1.22)	3	51.152	11.97728	-
Hypothetical protein_1	3	74.032	12.69183	-
C5a peptidase precursor (EC3.4.21.-)	3	86.929	14.04414	-
Iron compound ABC uptake transporter substrate-binding protein	3	37.72	14.30969	-
Neutral endopeptidase (EC3.4.24.-)	3	31.438	15.29397	-
Ribonucleotide reductase of class Ib (aerobic), beta-subunit (EC1.17.4.1)	3	38.858	15.49953	-
GTP-binding protein YqeH, required for biogenesis of 30S ribosome subunit	3	41.675	15.61347	-
UDP-N-acetylmuramoyl pentapeptide-lysineN(6)-alanyl transferase (EC2.3.2.10)	6	46.825	16.09414	-
Transcriptional regulator, AcrR family	3	21.032	16.34676	-

Na+/H+ antiporter	3	77.922	19.13114	-

S3: Ramachandran plot of chimeric-multiepitope protein vaccine predicted by PROCHECK. The plot of (A) 45F2 model indicated that 90.3%, , 8.5%, 0.7% and 0.7% of amino acid residues were located in most favored regions [A,B,L], additional allowed regions [a,b,l,p], generously allowed regions [~a,~b,~l,~p] and disallowed regions, respectively. While, the plot of (B) 42E2 zodel was represented at 83.0%, 11.6%, 4.8% and 0.7%, respectively.

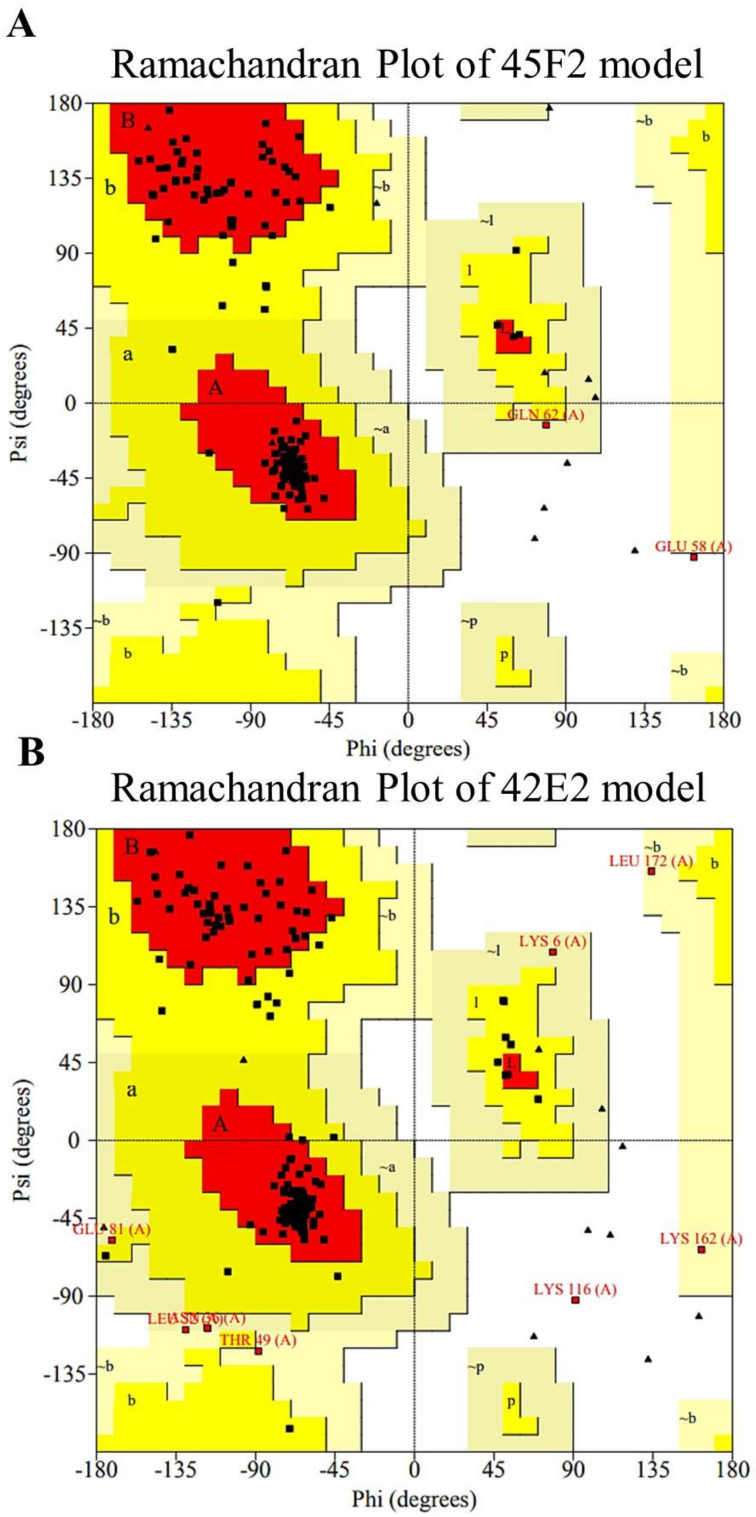

S4: Histogram of the codon-optimized chimeric-multiepitope 45F2 and 42E2. The codon quality distribution and codon quality plot of 45F2 (A-B) and 42E2 (C-D) showed percentage of codon that fell into a certain quality class. The CAI of 45F2 and 42E2 optimized gene are 0.92 and 0.93, respectively. The quality class means the CAI parameter >0.9 and equal 1 were considered as very good and perfect matching the codon usage preference of the target organism. The quality value of the most frequently used codon for a given amino acid in the desired the expression system is set to 100; codons with values less than 30 seem to diminish potential expression. The plots demonstrate the quality of the used codon at the indicated codon position. (E) and (F) represent GC content plots of 45F2 and 42E2 in a 40 bp window centered at the indicated nucleotide position. The ideal range of GC content should be in between 30% to 70%.

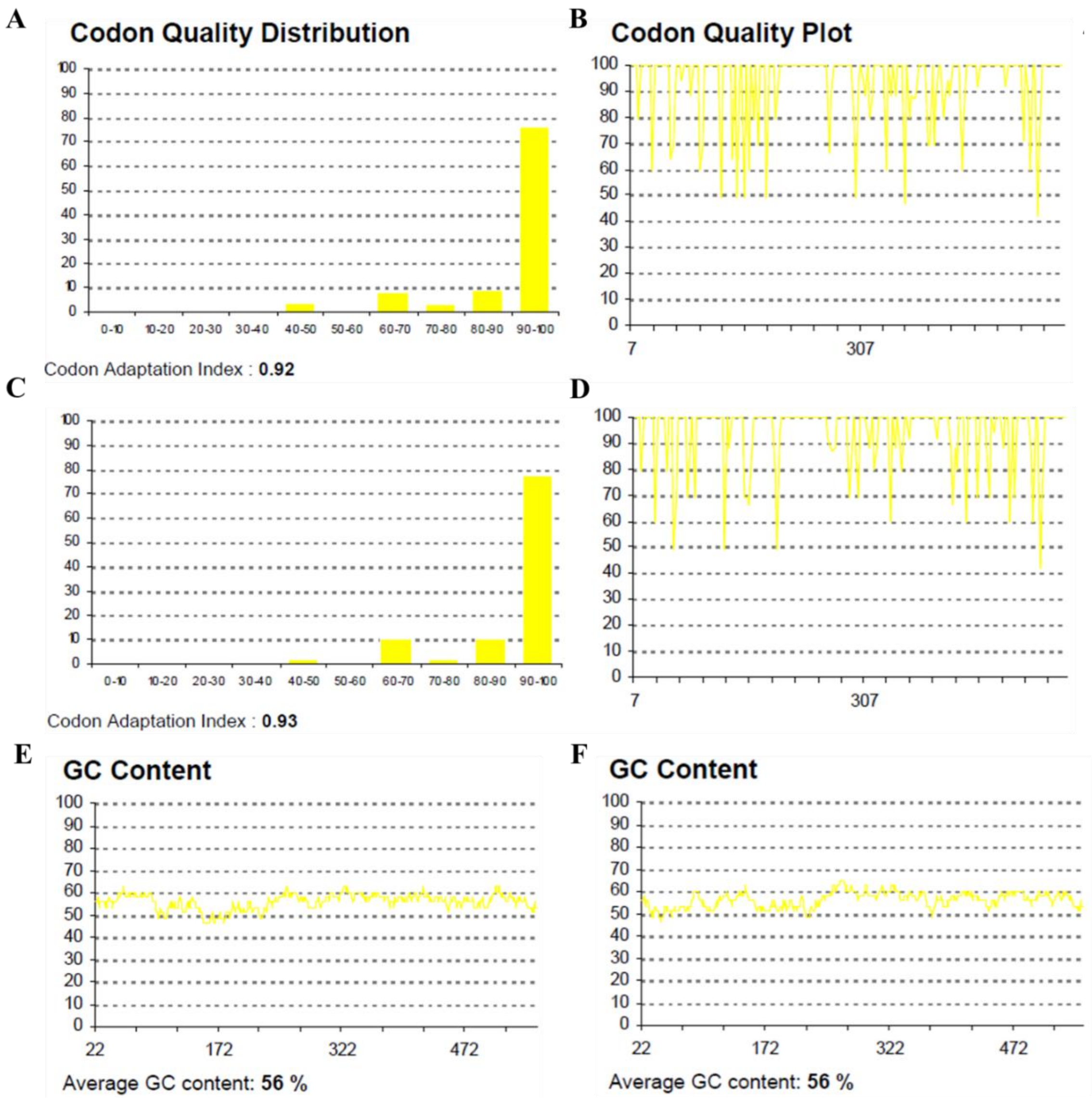

S5: The MFE secondary structure of chimeric-multiepitope vaccine candidates, 45F2 (A) and 42E2 (B). Regarding to base-pairing probabilities, the structure is colored by base-pairing probabilities. For unpaired regions, the color denotes the probability of being unpaired[51]. The MFE of 45E2 and 42E2 constructs represented the free energy of thermodynamic ensemble at -191.44 kcal/mol and -181.16 kcal/mol, respectively.

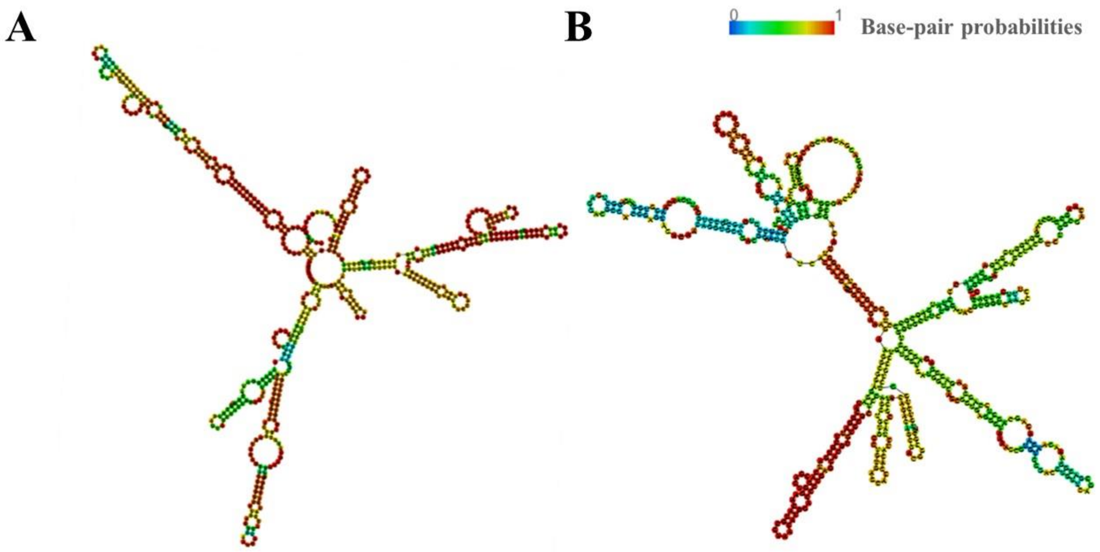

S6: Table of fish mortality during post-challenge for 21 days. The experimental groups were comprised of pcDNA3.1 as negative control, FKC as positive control, chimeric-multiepitope protein vaccine (42E2 and 45F2), as well as chimeric-multiepitope DNA vaccine (42E2 and 45F2) groups. #1, #2, and #3 indicated each replicate (n=10) of each treatment.

*The data were represented as cumulative mortality percentage in figure 6A.

**Date / Group**	**1**	**3**	**5**	**7**	**9**	**11**	**13**	**15**	**17**	**19**	**21**
pcDNA3.1 (#1)	0	0	4	4	6	6	6	7	7	7	8
pcDNA3.1 (#2)	0	1	5	5	5	5	5	5	6	6	6
pcDNA3.1 (#3)	0	2	4	5	6	6	6	6	7	7	7
FKC (#1)	0	1	1	1	1	1	1	1	1	2	2
FKC (#2)	0	0	0	0	0	0	0	0	0	0	0
FKC (#3)	0	1	2	2	2	2	2	2	2	2	3
42E2 protein (#1)	0	0	2	2	2	2	2	2	2	2	2
42E2 protein (#2)	0	1	2	2	3	3	3	3	3	3	3
42E2 protein (#3)	0	1	2	2	2	2	2	2	2	2	2
45F2 protein (#1)	0	0	1	1	1	1	1	1	1	1	2
45F2 protein (#2)	0	0	2	3	4	4	4	4	4	4	4
45F2 protein (#3)	0	0	2	2	3	3	3	3	3	3	3
42E2 DNA (#1)	0	0	0	0	0	0	0	0	0	0	0
42E2 DNA (#2)	0	2	3	3	3	3	3	3	3	3	3
42E2 DNA (#3)	0	0	0	1	2	2	2	2	2	2	2
45F2 DNA (#1)	0	0	1	1	2	2	2	2	2	2	2
45F2 DNA (#2)	0	0	1	1	1	1	1	1	1	1	1
45F2 DNA (#3)	0	0	2	2	2	2	2	2	2	2	2

S7: Bacterial isolation and clinical signs of challenged tilapia after challenge with *S. agalactiae*. Figure (A) represented PCR results of isolated bacteria from moribund fish after challenge. (M = VC 100 bp Plus DNA ladder, N = Negative, Ia and III = positive control of *S. agalactiae* serotypes Ia and III, 1-13 = Isolated bacteria from challenged fish). Figure (B) showed clinical signs of challenged fish. The left figure showed opaque eye (blue arrow) and the right figure showed abscess (yellow arrow).

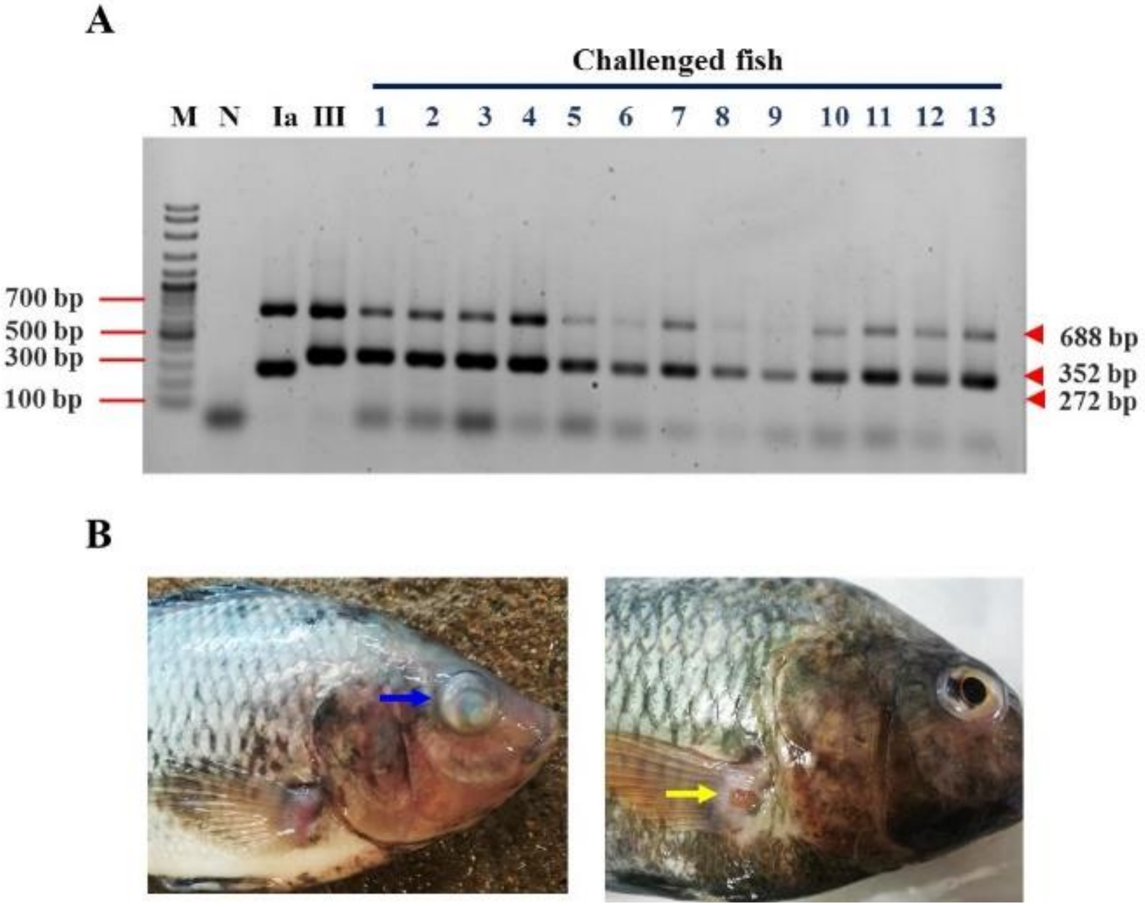

S8: Dot blot analysis of sera from fish immunized by pCDNA3.1 (control), chimeric recombinant protein vaccine 42E2 and 45F2 against whole cell *S. agalactiae* lysate serotype Ia and III.

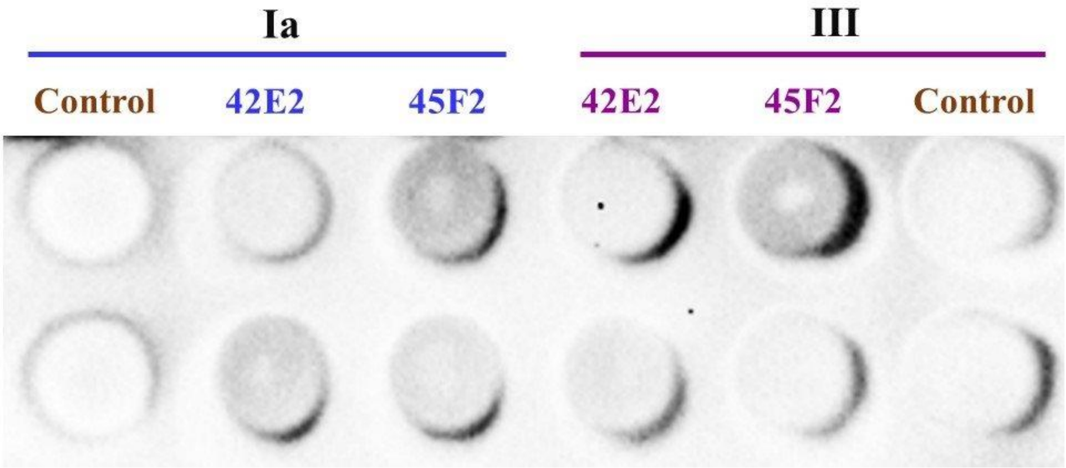

S9: Diagram of experimental designs for vaccine efficacy analysis.

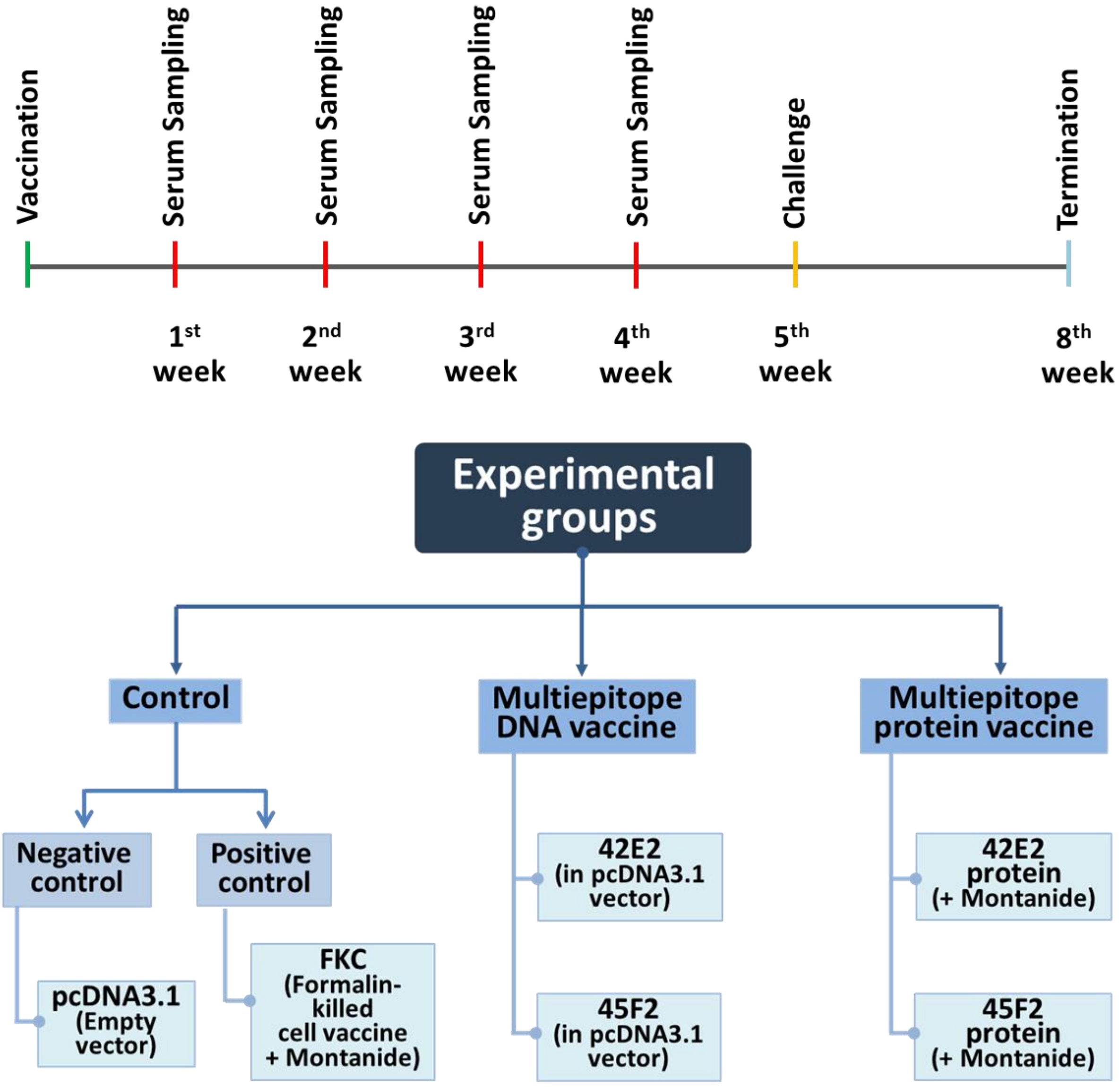

Supplement: Supplementary file 1 — Supplementary information [file 41598_2019_57283_MOESM1_ESM.pdf]
